# Proctalgia secondary to rectal arteriovenous malformation and inferior mesenteric vein stenosis in a patient post liver transplant

**DOI:** 10.1186/s42155-020-00196-1

**Published:** 2021-01-05

**Authors:** GM Healy, F Gondal, N Rutledge, DD Houlihan, JW McCann

**Affiliations:** 1 Department of Radiology, St Vincent’s University Hospital, D04 T6F4 Dublin, Ireland; 2 School of Medicine, University College Dublin, D04 V1W8 Dublin, Ireland; 3 Department of Hepatology, St Vincent’s University Hospital, D04 T6F4 Dublin, Ireland

**Keywords:** AVM, venous stenosis, embolization, proctalgia, rectum

## Abstract

**Background:**

Chronic proctalgia can have a major impact upon quality of life. There are many potential aetiologies however, in some patients no cause can be identified.

**Case presentation:**

We present a patient post liver transplant with intractable proctalgia, despite multidisciplinary management including opioids, nerve blocks and surgical intervention. An underlying rectal arteriovenous malformation (AVM) was subsequently identified and successfully treated with embolotherapy. The onset of symptoms coincided with the development of inferior mesenteric vein stenosis, likely leading to engorgement of the malformation due to impaired venous outflow. Neovascularisation secondary to the liver transplant procedure may also have contributed to growth of the lesion.

**Conclusion:**

This is a rare presentation of rectal AVM. These lesions can be treated with minimally invasive embolisation/sclerotherapy and should be considered in cases of unexplained proctalgia.

## Background

Proctalgia is a difficult clinical challenge which can cause significant impairment of quality of life. Common causes include malignancy, haemorrhoids, cryptitis and fissures. If no cause is found, it may be classified as a functional disorder, however it is important to thoroughly investigate and outrule treatable causes prior to this diagnosis.

We present a case of proctalgia secondary to rectal arteriovenous malformation (AVM). This is an unusual presentation for this condition, which more commonly causes gastrointestinal (GI) bleeding. We discuss the probable aetiologies for this patient’s symptoms, based upon review prior literature and describe our successful approach to endovascular management.

## Case presentation

A 54 year old man attended Liver Transplant clinic with severe proctalgia. He had undergone two liver transplant procedures for liver failure due to autoimmune hepatitis. Severe rectal and perineal pain, faecal urgency and intractable pruritus ani began two months after his second transplant. His pain persisted despite high dose opioids. Two nerve blocks were performed under fluoroscopic guidance by our local pain service, targeting the Ganglion Impar, a sympathetic ganglion located anterior to the sacrococcygeal joint which is involved with innervation of the coccyx, perineum and distal rectum (Gunduz and Kenis-Coskun 2017). The first block was performed with 5 mls of levobupivacaine 0.5% and 6.6 mg of dexamethasone. Pulsed radiofrequency ablation was performed at the second procedure, however there was no relief from pain.

Colonic biopsy was performed, demonstrating portal hypertensive colopathy, which is a common abnormality of the colonic mucosa in patients with portal hypertension, characterised pathologically by inflammatory change and vascular ectasia. It is usually asymptomatic, but can present with GI bleeding in up to 9% of cases (Rockey 2019). The patient’s case was discussed at multi-disciplinary rounds and the decision was made to attempt sigmoid proctocolectomy. However, at the time of laparotomy, extensive abdominal adhesions were identified and it was instead decided that a safer approach was to perform a diverting ileostomy, with the intention that diverting faeces from the rectum would improve symptoms. However, there was no subsequent change in symptoms.

**Fig. 1:**
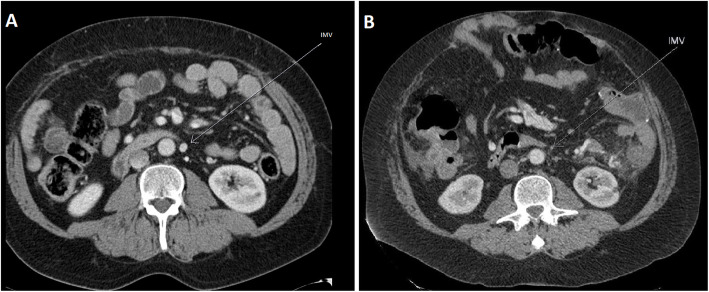
Contrast enhanced CT abdomen, portal venous phase. Demonstrating patent IMV at baseline (image **a**, Arrow) which had narrowed 7 years later (image **b**, Arrow). The patient underwent his first sclerotherapy treatment after image **b** was acquired.

Multiphasic Computed Tomography (CT) demonstrated two regions of abnormal, engorged vessels; at the antimesenteric border of the splenic flexure of the colon and adjacent to the rectum. There was arterial hypertrophy and arteriovenous shunting, compatible with arteriovenous malformations (AVM). The lesions did not have dominant draining veins, consistent with type IIa ‘Typical AVM nidus’ according to the Yakes’ Classification System (Yakes 2015). Upon review of prior imaging, it became apparent that the rectal AVM had been present for many years but that there had been progressive stenosis of the inferior mesenteric vein (IMV), with eventual segmental occlusion, coinciding with the development of proctalgia (Fig. 1. Image A and B were acquired seven years apart. The patient’s first sclerotherapy treatment was performed after image B was acquired.).

**Fig. 2:**
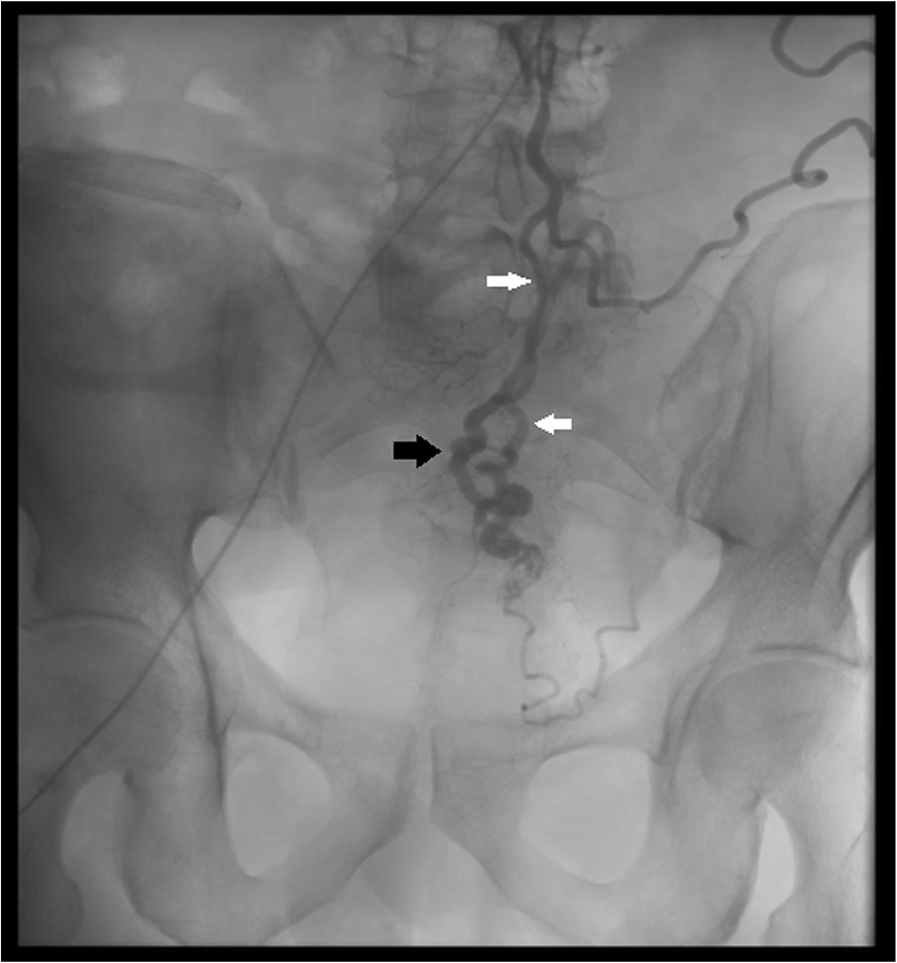
Fluoroscopic image demonstrating catheter injection of the Inferior Mesenteric Artery and superior rectal artery (white arrows). Early filling of veins is demonstrated (black arrows).

He was referred to Interventional Radiology (IR) and underwent angiography (Fig. 2) and sclerotherapy, using a 50:50 mixture of absolute alcohol and low-osmolar iodinated contrast introduced via a 2.4Fr 153 cm Rebar 18 microcatheter (Medtronic, Dublin, Ireland) superselectively into a superior rectal artery branch. This procedure was performed under General Anaesthesia. Excellent symptomatic response was reported, with improved quality of life. Proctalgia recurred three years later, requiring repeat sclerotherapy with resolution of symptoms. This has been maintained to date.

## Discussion

AVMs are tangles of poorly formed vessels where blood flows from arteries directly to veins, without an interposed network of capillaries. They can form anywhere in the body and may be congenital (sporadic or hereditary) or acquired (post trauma or iatrogenic). Small focal AVMs of the GI tract are often referred to as angiodysplasia. Reported population prevalence of small colonic AVMs on colonoscopy range from 0.8-3%, although they are uncommon in the rectum (Meyer et al. 1981; Höchter et al. 1985; Foutch et al. 1995). They typically present with episodic acute or occult GI bleeding. However, our patient did not experience this.

There are prior reports of ischaemic proctosigmoiditis or GI bleeding secondary to colorectal AVMs in the IMV territory (Ishikawa et al. 2020). However, to the best of our knowledge, this is the first report of severe intractable proctalgia secondary to an AVM, in the absence of colitis. Rectal AVMs have been reported in the setting of liver disease with portal hypertension, presenting as GI bleeding (Park et al. 2008), but there is no evidence that liver disease predisposes to the development of GI tract AVMs. Neovascularization is known to contribute to AVM expansion (Lu et al. 2011) and major abdominal surgery is a potent trigger of angiogenesis (Belizon et al. 2006), therefore the onset of symptoms may have been secondary to AVM growth in response to the liver transplantation procedure. However, impaired venous return due to progressive flow-related IMV stenosis and segmental occlusion leading to venous engorgement was probably the most significant precipitant of symptomatic deterioration. It has previously been demonstrated in cerebral AVMs that high flow through the lesion is associated with hyperplasia of the draining vein intima (Alqadi et al. 2019) and that the presence of venous outflow stenosis predisposes to development of AVM haemorrhage (Mansmann et al. 2000).

Treatment of AVMs depends on the degree of flow through the lesion, as well as the morphology, which can be described using Yakes’ classification (Yakes 2015). Endovascular or percutaneous angiography and embolisation/sclerotherapy is the first line of treatment for the majority of symptomatic AVMs (Funaki and Funaki 2016; Ishikawa et al. 2020). This usually involves accessing the feeding artery close to or at the abnormal connection between arterial and venous structures (the nidus) and injecting a sclerosant or embolic material. The goal is to occlude and obliterate the nidus, causing resolution of symptoms. A variety of agents can be used, including alcohol, Histoacryl, Ethylene vinyl alcohol copolymer or sodium tetradecyl sulfate (STS). Alcohol was chosen in our case because it is the most well established agent for the treatment of AVMs, with excellent long term outcomes (Mulligan et al. 2014; Khurana et al. 2018). The decision to use a 50:50 mixture of contrast and alcohol was made because vascular tortuosity prevented the positioning of the microcatheter tip immediately at the nidus. Because of this, there was concern for a potential risk of ischaemia in the normal tissues within the treated vascular territory. In such circumstances, a 50:50 mixture of alcohol and contrast is believed to protect normal tissues because the lower viscosity alcohol preferentially flows through the low resistance AVM channels, whereas the higher viscosity contrast flows through the normal vessels perfusing the adjacent tissues, a technique most suitable for treating type iv AVMs (Vogelzang et al. 2014; Yakes 2015).

If there is a dominant outflow vein (type IIB, IIIa or IIIB AVM lesions), immediate peri-nidal venous outflow occlusion with coils or other mechanical embolic devices is the recommended therapy, which may be performed alone or in addition to sclerotherapy (Yakes 2015). This was not feasible for this patient. Some patients may require surgical excision in combination with embolization.

## Conclusions

This case highlights a rare but important cause of proctalgia. AVM should be considered in cases of unexplained proctalgia because, if diagnosed, these lesions can be effectively treated by minimally invasive embolization. This case also demonstrates an interesting temporal association between the development of venous outflow stenosis and the onset of symptoms in AVMs, which is supported by prior literature in cerebral AVMs.

## Data Availability

Not applicable.

## Abbreviations

AVM: Arteriovenous malformation
CT: Computed Tomography
IMV: Inferior Mesenteric Vein
IR: Interventional Radiology
GI: Gastrointestinal
